# Improvement in Curcumin’s Stability and Release by Formulation in Flexible Nano-Liposomes

**DOI:** 10.3390/nano14221836

**Published:** 2024-11-17

**Authors:** Hua-Wei Chen, Su-Der Chen, Hung-Ta Wu, Chun-Hung Cheng, Chyow-San Chiou, Wei-Ting Chen

**Affiliations:** 1 Department of Chemical and Materials Engineering, National Ilan University, Yilan 260, Taiwan; hwchen@ems.niu.edu.tw (H.-W.C.); htwu@niu.edu.tw (H.-T.W.); aries730405a@gmail.com (C.-H.C.); 2 Department of Food Science, National Ilan University, Yilan 260, Taiwan; sdchen@niu.edu.tw; 3 Department of Environmental Engineering, National Ilan University, Yilan 260, Taiwan; cschiou@niu.edu.tw; 4 Department of Cosmetic Application & Management, St. Mary’s Junior College of Medicine, Nursing and Management, Yilan 266, Taiwan

**Keywords:** curcumin, liposome, particle size, encapsulation efficiency, thermal degradation, release

## Abstract

Curcumin is utilized extensively as Chinese medicine in Asia due to its antioxidant, antimicrobial, and inflammatory activities. However, its use has the challenges of low oral bioavailability and high heat sensitivity. The aim of this research was to produce flexible nano-liposomes containing curcumin using an innovative approach of ethanol injection and Tween 80 to enhance the stability and preservation of curcumin. The mean particle size, encapsulation efficiency, thermal degradation, storage stability, and curcumin release in flexible nano-liposomes were also investigated. We found that the mean particle size of curcumin-loaded flexible nano-liposome decreased from 278 nm to 27.6 nm. At the same time, the Tween 80 concentration increased from 0 to 0.15 wt%, which corresponded with the results of transmission electron microscopy (TEM) morphology analyses, and particle size decreased with an enhancement in Tween 80 concentration. Further, pure curcumin was quickly released within one hour at 37 °C, and first-order kinetics matched with its release curve. However, curcumin encapsulated in flexible nano-liposomes showed a slow release of 71.24% within 12 h, and a slower release pattern matched with the Higuchi model over 24 h, ultimately reaching 84.63% release. Hence, flexible nano-liposomes of curcumin made by a combination of ethanol injection and Tween 80 addition prevented the thermal degradation of curcumin, and enhanced its storage stability and preservation for future drug delivery applications.

## 1. Introduction

Curcumin, also called diferuloylmethane, is a key ingredient in curry powder widely used in Chinese medicine in Asia. Curcumin has demonstrated diverse pharmacological activities, including antioxidant [1], antimicrobial [2], anti-inflammatory [3], antirheumatic [4], anti-tumor [5], and immunomodulatory [6] effects. Despite its numerous benefits, the challenges of its low oral bioavailability and high heat sensitivity remain to be addressed to develop effective formulations [7,8]. The limited solubility of curcumin in water, particularly under acidic or neutral pH conditions, plays a vital role in these limitations. Additionally, the bioactive compounds in curcumin are susceptible to degradation during processing, which is influenced by factors such as the time, pH, and temperature [7,9].

Various encapsulation techniques can be employed, including liposomes [10], chelation strategies [11], microemulsion [12], and hydrogels [13], to enhance the bioavailability, thermal stability, and water solubility of curcumin. The biochemical structure of liposomes closely resembles that of the human cell membrane that ensures excellent biocompatibility and minimal irritation to the human body [14,15,16,17]. Hence, encapsulating low-solubility drugs in the liposomal bilayer increases their solubility and protects them from degradation, resulting in a significant enhancement in their therapeutic efficacy [10,18]. Previous studies have employed various methods to prepare liposomes, including reverse phase evaporation [19], ether or ethanol injection [20], probe sonication [21], thin film/handshaking [22], membrane extrusion [23], freeze–thaw [24], supercritical fluid [25], detergent dialysis [26], and high-pressure processing [27] (Table 1). The optimal size range for spherical liposomes in drug delivery applications is typically between 50 and 200 nm [28,29]. Several methods, including ether or ethanol injection, probe sonication, membrane extrusion, and freeze–thaw can produce small-size particles with a uniform distribution of liposomes. Detergent dialysis, reverse-phase evaporation, probe sonication, and membrane extrusion have been utilized in the laboratory-scale production of liposomes for drug encapsulation. However, only ethanol injection [20] and the supercritical fluid process [25] have been considered suitable and accessible for large-scale production.

Flexible liposomes, or ultradeformable liposomes, are composed of phospholipids and surfactants. Given the different geometric structures of surfactants and lipids, surfactants tend to have a bent structure, while lipid bilayers tend to be planar. Further, when surfactants enter the lipid bilayer, they reduce the bending energy of the bilayer. Compared to traditional liposomes, flexible liposomes exhibited a characteristic fluid membrane with high elasticity, facilitating the permeation of many molecules through the stratum corneum and cell membranes of the skin [30,31]. This property makes them suitable for transdermal drug delivery while minimizing the risk of drug leakage [32]. The surfactants in flexible liposomes reduced the interfacial tension of the bilayer that increased the bilayer fluidity in the stratum corneum and enhanced the permeability of liposomes encapsulating drugs into the skin [31,33]. Further, flexible liposomes easily reach the dermis and locally release the drug due to lysosomal degradation [34].

Food-grade synthetic surfactants, particularly Tweens, have been extensively employed for solubilization of curcumin [35]. The solubility of curcumin increased with longer chain lengths and reduced unsaturation in the surfactant tail groups [36]. Natural surfactants, such as casein, have also demonstrated effectiveness in solubilizing curcumin in aqueous solutions [37], making them potentially more suitable for specific food applications. A human feeding study investigated the pharmacokinetics of curcumin-fortified Tween 80 micelles. The curcumin-micelle formulation exhibited an area 185-fold higher under the curve than free curcumin, demonstrating enhanced bioavailability without any observed adverse side effects [36]. Given these findings, this research aimed at producing flexible nano-liposomes containing curcumin using an innovative approach involving ethanol injection and Tween 80 to enhance the curcumin stability and preservation. The mean particle size, size distribution, encapsulation efficiency of flexible nano-liposomes, and thermal degradation kinetics of curcumin were subsequently investigated.

## 2. Material and Methods

### 2.1. Preparation of Curcumin-Loaded Flexible Nano-Liposomes

All chemicals, including curcumin (98% purity; a mixture of bisdemethoxycurcumin, demethoxycurcumin, and curcumin; Acros Organics, Geel, Belgium), ethanol (Thermo Fisher Scientific, Waltham, MA, USA), Tween 80 (Acros Organics), cholesterol (Acros Organics), L-alpha-Lecithin (Acros Organics), and phosphate-buffered saline (PBS; Thermo Fisher Scientific), used in this study were reagent-grade reagents and employed without further purification. The curcumins were encapsulated by flexible nano-liposomes using the ethanol injection method with the modification of adding Tween 80. Further, 0.2, 0.4, or 0.6 mg/mL curcumin in various ratios of soy lecithin (8, 10, and 12 mg/mL) to cholesterol was dissolved (0.10, 0.15, and 0.20 mg/mL) in 10 mL ethanol. The required amount of curcumin (0.20 mg/mL) in the amount of L-alpha-lecithin (12 mg/mL) to cholesterol (0.15 mg/mL) was dissolved in ethanol. The solution obtained was injected into 0.1 wt% Tween 80 solution in 60 mL PBS for the formation of curcumin-loaded flexible nano-liposomes at a pH of 7.4 ± 0.2. The residual ethanol in the solution of curcumin-loaded liposomes and curcumin-loaded flexible liposomes was completely evaporated by rotary evaporation at room temperature and constant speed for 40 min. Curcumin-loaded flexible nano-liposomes were formed after residual ethanol was vapored in the magnetic stirrer for 40 min at room temperature.

### 2.2. Particle Size Measure

The dried nano-liposome samples were analyzed by transmission electron microscopy (TEM, HITACHI, HT7700, Chiyoda, Japan) and the morphology of liposomes was imaged. The zeta potential for nano-liposomes was measured by a Malvern ZetaSizer (DKSH, Zurich, Switzerland). The mean particle size and the polydispersity index (PDI) of nano-liposomes were determined by a zeta/nanoparticle analyzer (Zetasizer Nanoplus-3, Worcestershire, UK) using the autocorrelation functions with a refractive index of 1.33.

### 2.3. Encapsulation Efficiency and Thermal Stability of Curcumin Liposome

The concentration of free curcumin passing through the ultra-centrifugal filter tube (molecular weight or MW cutoff of 100 kDa, Microsep^®^, Johannesburg, South Africa) and the encapsulation efficiency of the nano-liposomes were detected by a UV/VIS spectrophotometer (INESA, 752N, Shanghai, China) after the samples were centrifuged at 7000 RCF and 4 °C for 10  min (Hermle Centrifuge Z326K, Gosheim, Germany), as reported in previous research [10,38]. The thermal stability and release of curcumin-loaded flexible nano-liposomes in PBS was analyzed by a UV/VIS spectrophotometer at different temperatures, while the UV/VIS wavelength of curcumin was chosen as 425 nm for the detection of curcumin. The stability of curcumin was determined by the ratio of initial concentration and concentrations of curcumin at each sampling time point. Pure curcumin and curcumin-loaded flexible nano-liposomes were added into a 100 mL phosphate-buffered solution to evaluate the curcumin release at each sampling time point. The samples were covered with aluminum foil to protect them from light exposure in a 37 °C (body physiological temperature) incubator.

## 3. Results and Discussion

### 3.1. Morphology Analysis of Flexible Nano-Liposomes by Transmission Electron Microscopy

**Figure 1:**
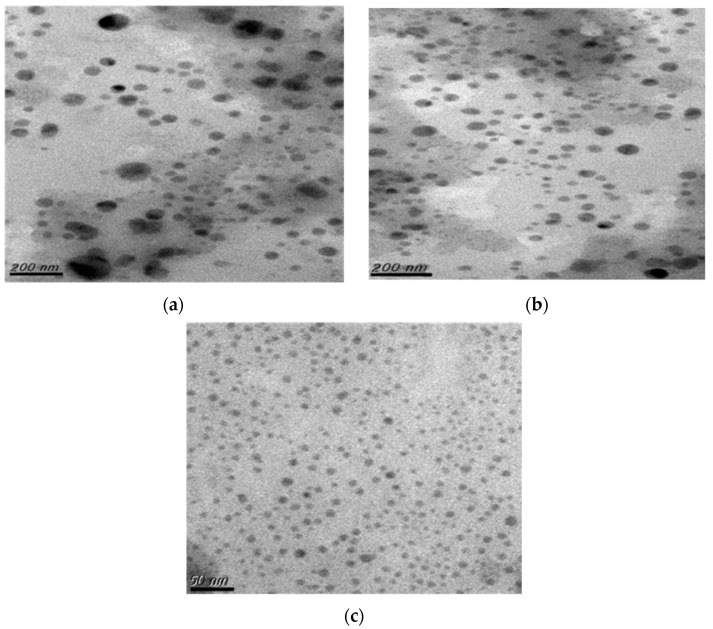
Transmission electron microscopy (TEM) images of flexible nano-liposomes loaded with curcumin prepared with different Tween 80 concentrations (**a**) 0.00 wt% (**b**) 0.05 wt% (**c**) 0.15 wt% (soy lecithin concentration: 12 mg/mL; cholesterol concentration: 0.15 mg/mL; curcumin concentration: 0.20 mg/mL).

**Figure 2:**
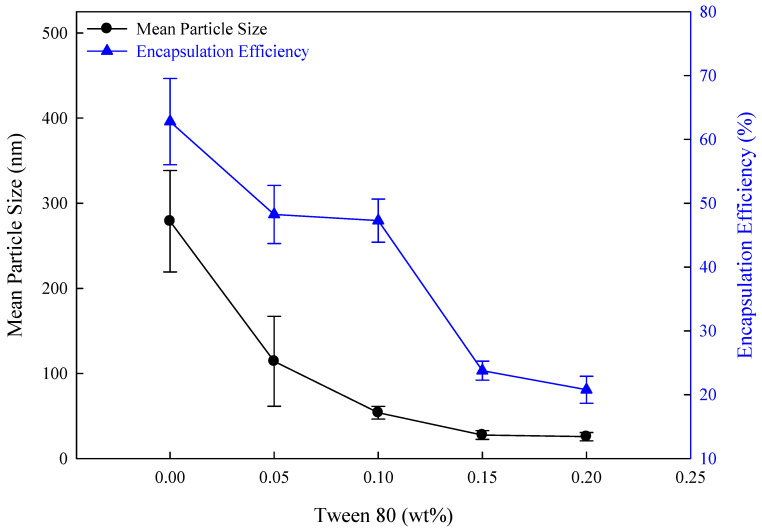
The effect of different Tween 80 concentrations on flexible nano-liposomes (soy lecithin concentration: 12 mg/mL; cholesterol concentration: 0.15 mg/mL; curcumin concentration: 0.20 mg/mL).

The morphology of flexible nano-liposomes prepared in different Tween 80 concentrations was observed by TEM, as shown in Figure 1. Flexible nano-liposomes mainly possessed spherical and spherical-like vesicle structures suitable for encapsulating drugs regardless of the size of the vesicles. The mean particle size decreased from 200–300 nm to 15–30 nm as the Tween 80 concentration increased from 0 wt% to 0.15 wt%. Based on the size of the nano-liposomes with a zeta/nanoparticle analyzer, the mean particle size of liposomes produced without Tween 80 addition was the largest (278.8 nm), as shown in Figure 2. The mean particle size of flexible nano-liposomes decreased from 114.2 nm to 27.6 nm as the Tween 80 concentration increased from 0.05 wt% to 0.15 wt%. These results were also observed in the TEM morphology analyses. As Tween 80 acts as a surfactant, it can reduce the interfacial tension of the phospholipid bilayer and increase the phospholipid bilayer fluidity to prepare smaller particle sizes for enhanced drug permeability to the skin [39].

The surfactant tends to have a curved structure due to the difference in the geometric structure of the surfactant and the lipid. This makes the lipid bilayer highly elastic; when subjected to a shear force homogeneously, the particle size can be effectively reduced. In a previous report, dimethyl glycyrrhizinate (DPG) was added to traditional liposomes as an anionic surfactant to effectively reduce the particle size from 375.6 nm to 197.2 nm owing to the decrease in the surface tension [33]. According to another report, when the liposome size was of less than 100 nm, it could penetrate the choroidal barrier and reach the retinal pigment epithelial cells [40].

### 3.2. Zeta Potential Analysis of Flexible Nano-Liposomes

**Figure 3:**
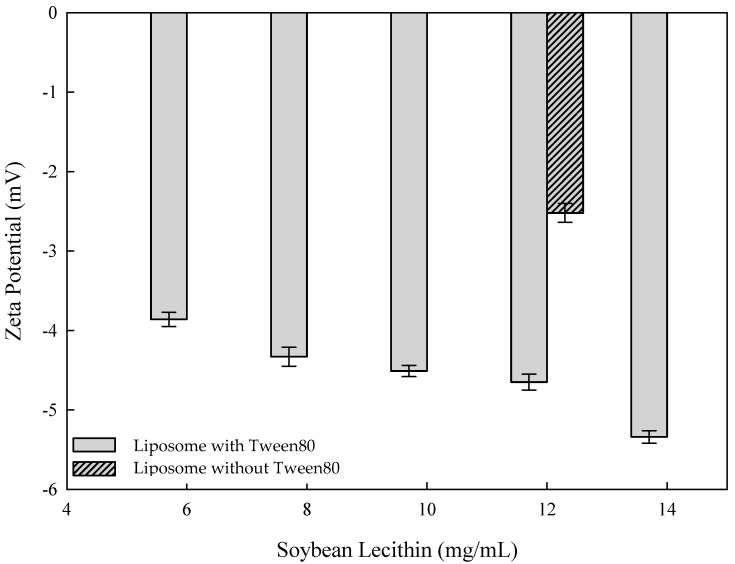
Zeta potential of various liposome contents with and without Tween 80 (soy lecithin concentration: 12 mg/mL; cholesterol concentration: 0.15 mg/mL; curcumin concentration: 0.20 mg/mL; Tween 80 concentration: 0.1 wt%).

Liposomes with a lipid bilayer structure are mainly composed of soy lecithin, organic solvents (such as ethanol, isopropyl alcohol, methanol, and diethyl ether), and cholesterol. It is to be noted that soy lecithin has a negatively charged surface. The effect of different concentrations of soybean lecithin and Tween 80 on the zeta potential of liposomes is shown in Figure 3. The zeta potential decreased from −3.86 mV to −5.34 mV as the concentration of soybean lecithin gradually increased from 6 mg/mL to 14 mg/mL. The decrease in zeta potential can be attributed to the phosphate group (PO_4_^3−^) of soybean lecithin. Research by Toledo et al. revealed that the negative value of the zeta potential of the liposome is related to the ionization of carboxyl and phosphate groups [41]. This is because lecithin is ionized into carboxyl and phosphate groups in an aqueous solution, which makes the potential negative. Zeta potential represents the surface charge of the liposome, which shows that the surface of lipid bilayers acquires electric charge; so, the electrostatic repulsion from the liposome’s surface interrupts the liposome’s intermolecular cohesion to reach the stability effect of the liposome.

According to an earlier study by Alomrani et al. (2019), chitosan-coated liposomes were used as carriers to enhance the antitumor efficacy of 5-fluorouracil against colorectal cancer [42]. The addition of Tween 80 resulted in lower zeta potential values (−7.5 mv), while higher zeta potential values were seen without the addition of Tween 80 (−2.3 mv) [42]. As Tween 80 is an ethylene oxide polymer composed of hydrophilic polyethylene groups, it has many negative charges of oxygen atoms. Therefore, the zeta potential became negative after adding Tween 80 to liposome samples. As evidenced by negative charge values, Tween 80 was added to liposome samples in this study. The variation in soybean lecithin was used to determine the effect of zeta potential on liposomes after adding Tween 80. Hence, it was expected that the zeta potential value would increase by increasing the addition of soy lecithin [18]. Therefore, this study aimed to analyze the charge of one of the constituents of liposomes (soy lecithin) to determine the effect of zeta potential on the stability of liposome samples.

### 3.3. Evaluation of Heat-Sensitive Properties of Curcumin

Curcumin has been proven to possess various therapeutic effects, including antioxidant, anti-inflammatory, antibacterial, and anticancer activities. However, a limitation is that curcumin is sensitive to environmental temperature and light. Therefore, in this study, a curcumin aqueous solution (curcumin), curcumin liposomes (Liposome without Tween80), and flexible nano-liposomes of curcumin (Liposome with Tween80) were subjected to a thermal sensitivity test for 12 h at 4 °C, 25 °C, and 40 °C.

**Figure 4:**
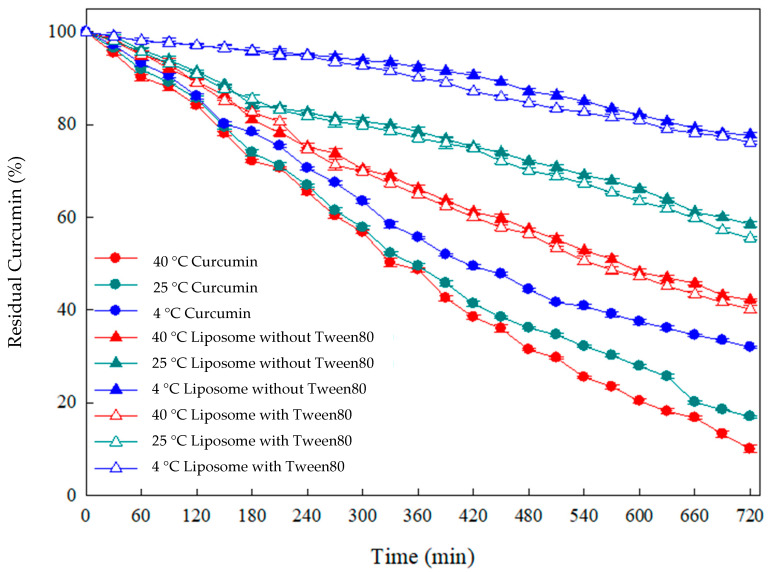
Degradation of curcumin at different temperatures with or without encapsulation in flexible nano-liposomes (soy lecithin concentration: 12 mg/mL; cholesterol concentration: 0.15 mg/mL; curcumin concentration: 0.20 mg/mL; Tween 80 concentration: 0.1 wt%).

After 720 min of incubation, the curcumin degradation in the aqueous solution was 68.0%, 82.8%, and 89.35% at 4 °C, 25 °C, and 40 °C, respectively, as shown in Figure 4. The degradation of curcumin became more pronounced with increasing the environmental temperature. This decline in stability can be attributed to the poor solubility of curcumin in aqueous solutions and its hydrolytic degradation under conditions of thermal processing. Notably, a study by Suresh et al. highlighted that ferulic acid, vanillin, and vanillic acid were the primary intermediates in curcumin degradation during heat treatment, primarily due to the vulnerability of the diketone bridge in its molecular structure [8]. Moreover, the influences of the method and storage temperature were evaluated on the physical and chemical stability of the prepared nanodispersions. The results obtained indicated that prepared transparent nanodispersions maintained their physical and chemical stability at 4 and 25 °C for 4 months. The results also showed that the prepared nanodispersions using subcritical water (temperature of 120 °C and pressure of 1.5 atm for 2 h) had a higher stability (minimum changes in the particle size) and lower curcumin loss (0.36%) [43].

The degradation rate showed a decreasing trend by encapsulated curcumin with liposomes and flexible liposomes. The degradation of curcumin in liposomes and flexible nano-liposomes after storing for 720 min at 4 °C was 22.14% and 23.88%, respectively. Furthermore, the degradation of curcumin was 41.51% and 44.51%, respectively, even when stored at 40 °C for 720 min. Thus, these results demonstrated that encapsulation with liposomes or flexible liposomes could markedly improve the stability of curcumin and delay its degradation. This improvement is because the keto-enol groups were effectively shielded within the hydrophobic core of the liposomes. According to an earlier report, curcumin molecules interact with the double-carbon chains of phosphate groups and phosphatidylcholine, allowing the two phenoxyl groups of curcumin to be enclosed in the liposomal hydrophobic core, enhancing curcumin stability [44].

### 3.4. Storage Stability of Curcumin

**Figure 5:**
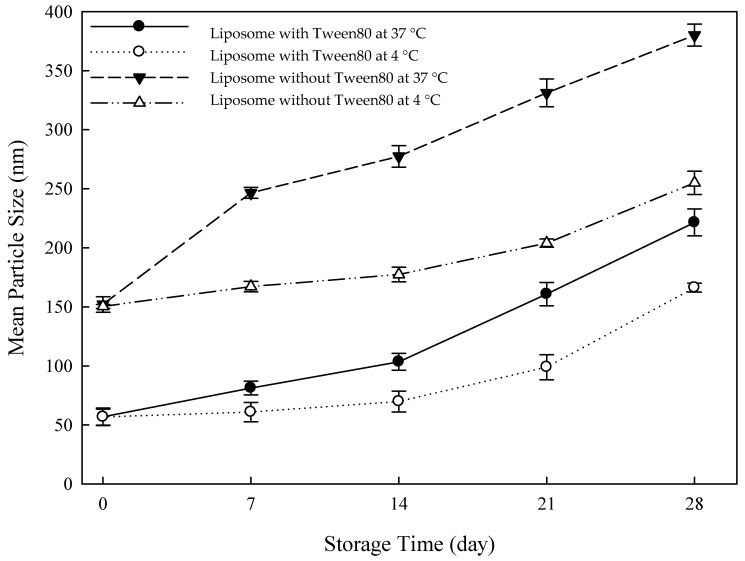
Effects of different temperatures on curcumin liposomes and curcumin-loaded flexible nano-liposomes (soy lecithin concentration: 12 mg/mL; cholesterol concentration: 0.15 mg/mL; curcumin concentration: 0.20 mg/mL; Tween 80 concentration: 0.1 wt%).

Curcumin liposomes (Liposome without Tween80) and flexible nano-liposomes of curcumin (Liposome with Tween80) were stored at 4 °C and 37 °C, and their mean particle sizes were then measured in a stability test for 28 days. As observed in Figure 5, both curcumin liposomes and curcumin flexible nano-liposomes exhibited larger mean particle sizes at the higher temperature of 37 °C than those stored at the lower temperature of 4 °C. This difference was attributed to the increased temperature, causing a reduction in the stability of the liposomal bilayer. The higher temperature results in greater fluidity of the liposomal bilayer, which causes phospholipid molecules to detach from the bilayer and accordingly disrupt the original structural of phospholipid molecules. Previous results also proposed that the increase in the liposomal mean particle size was caused by the encapsulation of 5-fluorouracil at a higher temperature [42].

It can be observed that the mean particle sizes of both curcumin liposomes and flexible nano-liposomes increased with the extension of storage time at 4 °C and 37 °C, as shown in Figure 5. This observation is due to the spontaneous rearrangement of phospholipid molecules in the bilayer of curcumin liposomes, leading the liposomes toward a more stable configuration. This rearrangement results in an increase in the mean particle size of curcumin liposomes from 152.1 nm to 380.1 nm over 28 days. Flexible nano-liposomes demonstrated increased flexibility of the liposomal bilayer owing to the presence of surfactants. The mean particle size of flexible nano-liposomes increased from 56.9 nm to 221.5 nm. This reduction in size was attributed to the action of the edge activator (Tween 80) on the membrane of liposomes to reduce the surface tension of the media, leading to phospholipid arrangement in small vesicles [42]. In summary, flexible nano-liposomes, exhibited significantly smaller mean particle sizes than conventional curcumin liposomes, regardless of storage at 37 °C or 4 °C. This result demonstrated the importance of Tween80 (Polysorbate 80), as it substantially reduced particle size and stabilized the structure of the liposomes.

### 3.5. Release of Curcumin from Flexible Nano-Liposomes

**Figure 6:**
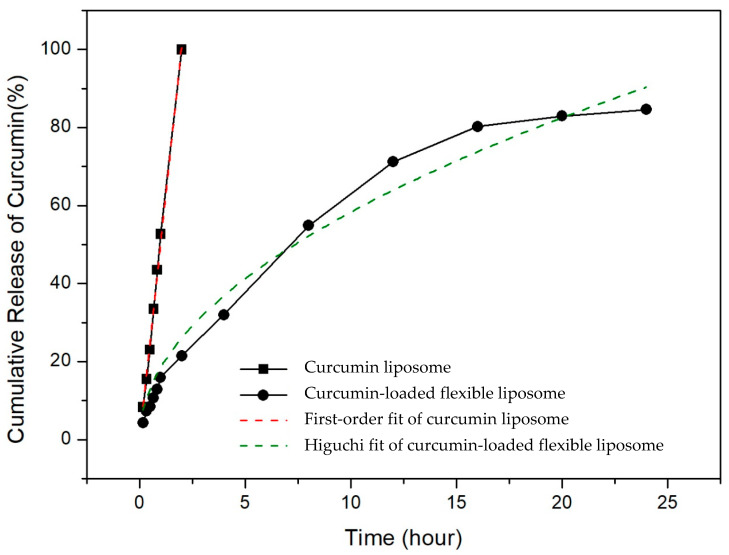
Effects of different temperatures on curcumin liposomes and curcumin-loaded flexible nano-liposomes (soy lecithin concentration: 12 mg/mL; cholesterol concentration: 0.15 mg/mL; curcumin concentration: 0.20 mg/mL; Tween 80 concentration: 0.1 wt%).

The extremely short half-life and rapid metabolism of curcumin necessitate frequent dosing to maintain a therapeutic drug concentration. This study explored the effect of liposomes on curcumin release to enhance the stability of curcumin and prolong its release, thereby improving its bioavailability. Pure curcumin quickly released within 1 h at 37 °C, as shown in Figure 6. However, curcumin encapsulated in flexible nano-liposomes showed a slow release of 71.24% in 12 h, followed by a slower release over 24 h, and ultimately reached 84.63% release. The kinetics of zero-order, first-order, Higuchi, and second-order equations for pure curcumin and flexible nano-liposomes were considered to describe the release behavior of curcumin. Higuchi models were employed for the prediction of the permeability in nanocomposite membranes with randomly distributed spherical particles in a polymer matrix [44]. Moreover, the Higuchi equation is a mathematical model of drug release originally conceived to describe the release of a drug from a thin ointment film into the skin. Among other considerations, the model assumes that the initial drug concentration in the film is much higher than the solubility of the drug in the ointment base. Hence, drug release will encompass both drug dissolution and diffusion. Moreover, it is assumed that the dissolution of the drug within the ointment base will be rapid in comparison with its diffusion [45]. Based on the best coefficients of determination (*R*^2^), the release behavior of free curcumin followed first-order kinetics where the *R^2^* was 0.983. However, the mechanism of diffusional release of flexible nano-liposomes mainly tended to follow the Higuchi model with *R^2^* values of 0.996. This extended release is primarily attributed to the diffusion and dissolution of curcumin being influenced by the phospholipid bilayer. In an earlier study, it was found that curcumin rapidly released within 120 min at 23 °C and the release time extended to 360 min when encapsulated in liposomes [46]. Thus, the flexible nano-liposomes demonstrated a delayed drug release and increased curcumin bioavailability in comparison to pure curcumin.

### 3.6. Effect of Tween 80 Concentration in Flexible Nano-Liposomes

A liposome that measures less than 80 nm in size can penetrate the surface layer of the skin to the deep part of the skin (dermis). Further, this depth is twice that of the liposome with a particle size larger than 100 nm [40]. The mean particle size and encapsulation efficiency of flexible nano-liposomes after adding Tween 80 in various concentrations is shown in Figure 2.

The mean particle size of liposomes was 278.8 nm without any Tween 80 addition (0 wt%). Further, the mean particle size decreased from 278.8 nm to 25.7 nm but the encapsulation efficiency decreased from 62.78% to 20.78% as the Tween 80 concentration increased from 0 wt% to 0.20 wt%. Further, the mean particle size reduced to below 80 nm with the benefits of drug delivery and high skin absorption when Tween 80 was added at 0.10 wt%, 0.15 wt%, and 0.20 wt%. However, reducing the average particle size tends to lead to a smaller space for encapsulating curcumin, resulting in a lower encapsulation efficiency (around 20.78%) when Tween 80 was added in excess (0.20 wt%). According to these results, preparing flexible nano-liposomes with both small mean particle size and high encapsulation efficiency is impossible. The addition of 0.1 wt% Tween 80 is an appropriate method to produce nano-liposomes with a low particle size (<80 nm) and relatively high encapsulation efficiency for effective penetration of the skin and drug delivery.

### 3.7. Effect of Soy Lecithin Concentration in Curcumin Flexible Nano-Liposomes

**Figure 7:**
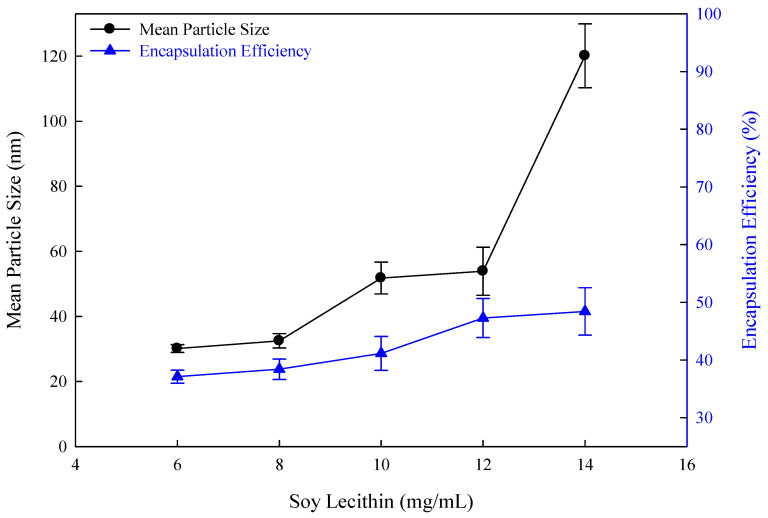
The effect of soy lecithin concentrations on flexible nano-liposomes (cholesterol concentration: 0.15 mg/mL; curcumin concentration: 0.20 mg/mL; Tween 80 concentration: 0.1 wt%).

Liposomes can simultaneously load both hydrophilic and lipophilic drugs because they are composed of phosphate groups and two hydrophobic fatty acid chains. As the concentration of soy lecithin increased from 6 mg/mL to 14 mg/mL, the mean particle size increased from 30.11 nm to 120.1 nm, and the encapsulation efficiency increased from 37.11% to 48.42% (Figure 7). The mean particle size of flexible nano-liposomes also increased with the increase in the soy lecithin concentration. The lipid bilayer becomes thicker with the addition of soy lecithin, increasing the size of flexible nano-liposomes. When the flexible nano-liposome particle size is increased, the space of the lipid bilayer inside the liposome expands, increasing the volume available for encapsulating lipophilic drugs. The encapsulation efficiency subsequently increases with the increasing content of soy lecithin. In an earlier report, soy lecithin was used to encapsulate eugenol to improve the photosensitivity and antibacterial properties of eugenol [20]. It was found that as the soy lecithin concentration increased from 10 mg/mL to 30 mg/mL, the particle size of the liposomes increased from 210 nm to 356 nm, similar to the results of other researchers [20]. Due to the proportional relationship between the size of liposomes and soy lecithin concentration, the size of flexible nano-liposomes was of less than 80 nm for drug delivery to the skin as the concentration of soy lecithin was lower than 12 mg/mL.

### 3.8. Effect of Cholesterol Concentration in Flexible Curcumin Nano-Liposomes

**Figure 8:**
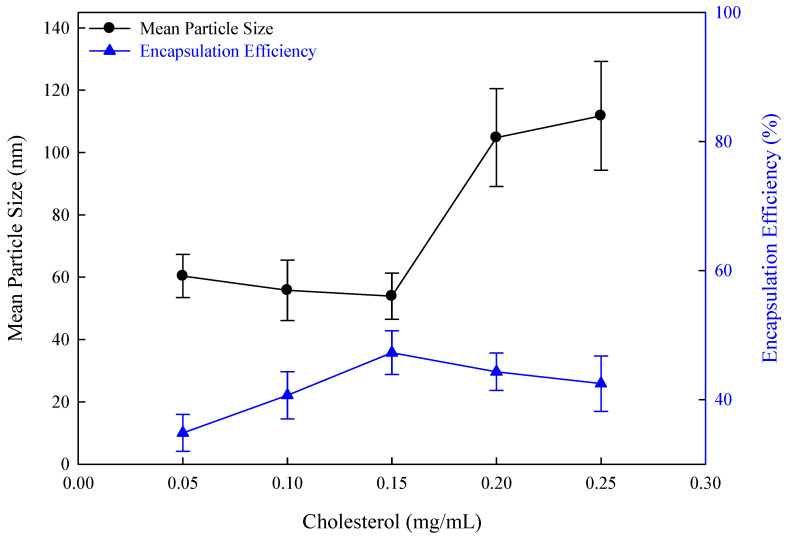
The effect of cholesterol concentrations on flexible nano-liposomes (soy lecithin concentration: 12 mg/mL; curcumin concentration: 0.20 mg/mL; Tween 80 concentration: 0.1 wt%).

Cholesterol can regulate the fluidity of lipid bilayers, leading to a tighter arrangement of lipid bilayers and reducing drug leakage. The effect of different cholesterol concentrations on the mean particle size and encapsulation efficiency in this study is shown in Figure 8. As the cholesterol concentration increased from 0.05 mg/mL to 0.15 mg/mL, the mean particle size decreased from 60.4 nm to 54.1 nm, while the encapsulation efficiency increased from 34.85% to 47.28%. At low cholesterol concentrations, the hydrophobic fatty acid chains were dispersed, enhancing the fluidity of the lipid bilayer in flexible nano-liposomes and producing smaller particle sizes. The presence of a small amount of cholesterol in the lipid stabilized the structure of the lipid bilayer, preventing drug leakage and enhancing encapsulation efficiency. When the cholesterol concentration was gradually increased from 0.15 mg/mL to 0.25 mg/mL, the mean particle size increased from 54.1 nm to 111.8 nm due to the lipid bilayer rigidity in flexible nano-liposomes. Simultaneously, the encapsulation efficiency decreased gradually, and curcumin delivery was blocked owing to rigidity when the cholesterol concentration exceeded 0.15 mg/mL. The alkyl chain of cholesterol interacted with the phospholipid head group upon excessive cholesterol addition, causing the insertion of cholesterol into the phospholipid bilayer membrane and the production of larger-sized microspheres [47,48]. Therefore, adding an optimal concentration of cholesterol (0.15 mg/mL) stabilized the phospholipid bilayer structure to reduce drug leakage and generated smaller-sized flexible nano-liposomes.

### 3.9. Effect of Curcumin Concentration in Flexible Nano-Liposomes

**Figure 9:**
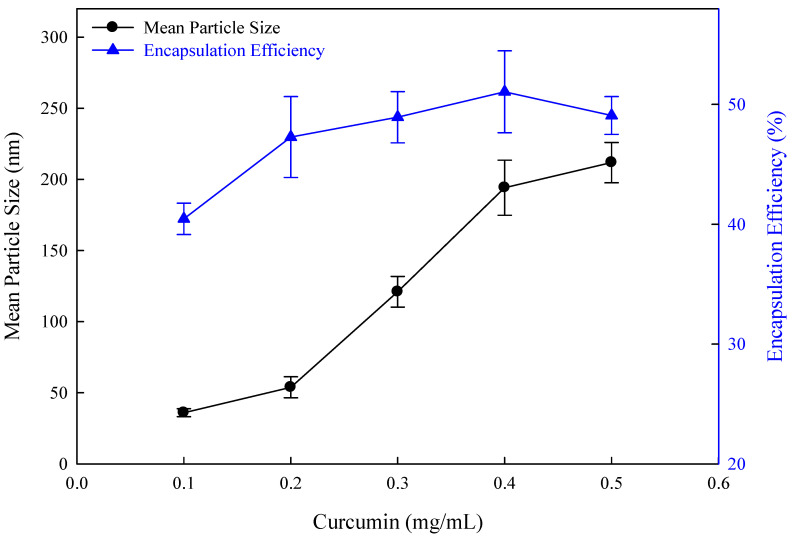
The effect of curcumin concentrations on curcumin-loaded flexible nano-liposomes (soy lecithin concentration: 12 mg/mL; cholesterol concentration: 0.15 mg/mL; Tween 80 concentration: 0.1 wt%).

The encapsulation of the lipophilic drug curcumin within the phospholipid bilayer of liposome can address its challenges, such as its low solubility, susceptibility to degradation, and poor bioavailability. As shown in Figure 9, the extent of curcumin encapsulation significantly influenced the mean particle size and encapsulation efficiency. As the concentration of curcumin gradually increased from 0.1 mg/mL to 0.5 mg/mL, the mean particle size of liposomes increased from 36.0 nm to 211.8 nm. This increase was attributed to the incorporation of curcumin into the lipid bilayer, resulting in a larger particle size. However, when the curcumin concentration was increased from 0.4 mg/mL to 0.5 mg/mL, the excessive curcumin molecules could not enter the interior of the flexible nano-liposome, decreasing the encapsulation efficiency. Other studies also propose that the overloading of curcumin led to a significant increase in particle size and a decrease in encapsulation efficiency [49,50]. It was proposed that the hydrophobic curcumin influenced the hydrophobic interactions of the phospholipid acyl chains and caused the leakage of the encapsulated substance.

The mean particle size of liposomes was 2.0 × 10^3^ nm, 160–210 nm, 147.8 nm, 969 nm, 100–320 nm, 150−200 nm, and 240.7 nm by reverse-phase evaporation [19], ether or ethanol injection [20], probe sonication [21], thin film/handshaking [22], membrane extrusion [23], supercritical fluid, depressurization of an expanded liquid organic solution (DELOS) [25], and ethanol injection with high-pressure processing (HPP) [27], respectively. The mean particle size of flexible nano-liposomes obtained by ethanol injection with surfactant in this study was significantly smaller than that obtained with other preparation methods, as shown in Table 1. The nano-sized particle dimensions in this study show potential for drug delivery and industrial uses, such as cosmetic, dairy beverage, and pharmaceutical industries. The benefit of practical application is that nano-sized liposomes can be prepared easily and uniformly for large-scale production.

## 4. Conclusions

The mean particle size of curcumin-loaded flexible nano-liposomes decreased from 114.2 nm to 27.6 nm as the addition of Tween 80 increased from 0.05 wt% to 0.15 wt%, and this pattern was also consistent with the results of TEM morphology studies. Additionally, as the concentration of soybean lecithin gradually increased from 6 mg/mL to 14 mg/mL, the zeta potential decreased from −3.86 mV to −5.34 mV. Hence, the reason for this zeta potential decrease was attributed to the phosphate group (PO_4_^3−^) of soybean lecithin. The results revealed that flexible nano-liposomes (liposomes with Tween 80) effectively reduced the thermal degradation of curcumin after 720 min of testing at 4 °C, 25 °C, and 40 °C, and the degradation of curcumin was in the order of free curcumin >curcumin-loaded liposomes (Liposome without Tween 80) >flexible nano-liposomes. Furthermore, the mean particle size of curcumin liposomes (Liposomes without Tween 80) increased from 152.1 nm to 380.1 nm during 0 to 28 days of storage tests. However, the mean particle size of flexible nano-liposomes (Liposomes with Tween 80) increased from 56.9 nm to 221.5 nm during 0 to 28 days of storage tests because the enhanced flexibility caused the fusion of vesicles among flexible nano-liposomes. However, the encapsulation efficiency decreased if the cholesterol concentration exceeded 0.15 mg/mL, and drug delivery was blocked owing to the rigidity of the liquid bilayer. The maximum addition of curcumin permissible was 0.4 mg/mL, as, if the curcumin concentration increased from 0.4 mg/mL to 0.5 mg/mL, the excessive curcumin molecules were not able to enter the interior of flexible nano-liposome, which decreased the encapsulation efficiency. Therefore, the curcumin-loaded flexible nano-liposomes prepared by combination of ethanol injection and Tween 80 addition showed increased storage stability and protection of curcumin, and exhibited a slow-release effect suitable for application in the cosmetic, dairy beverage, and pharmaceutical industries.

## Figures and Tables

**Table 1:** Comparison of various methods of liposome preparation.

Method	Advantages	Disadvantages	Particle Size (nm)	Encapsulation Efficiency (EE) (%)
Reverse phase evaporation [19]	High encapsulation efficiency (EE; >65%)	Complex preparation process	2.0 × 10^3^	-
Ether or ethanol injection [20]	Simple preparation process, no harmful substances, and high EE	The removal of organic solvents is difficult in large-scale production	160–210	65.4
Probe sonication [21]	Single-layered and smaller-sized liposomes	Oxidation of lipids and deposition of metal debris at the probe tip	147.8	44.02
Thin film/hand shaking [22]	Easily accessible equipment and simple preparation process	Potential safety concerns due to residual chloroform	969	67.34
Membrane extrusion [23]	Uniform distribution of particle size	More complex preparation process	100–320	-
Freeze–thaw [24]	Uniformly sized single-layer liposomes	Lower EE (approximately 20–30%)	2.6–8.2 × 10^3^	20–30
Supercritical fluid, DELOS [25]	Smaller-sized liposomes, uniform distribution, and large-scale production	Low EE of hydrophilic compounds in liposomes	150−200	20–50
Detergent dialysis [26]	Easily accessible equipment	Requires a large amount of organic solvents (hazardous health)	-	-
Ethanol injection with HPP [27]	Environmentally friendly, uniform distribution, and large-scale production	Requires a high-pressure equipment	240.7	77.8
Ethanol injection with surfactant (this study)	Nano-sized liposomes, uniform distribution, and large-scale production	Low EE	20–300	20.7–62.7

## Data Availability

The original contributions presented in this study are included in this article; further inquiries can be directed to the corresponding author.
